# Protocol to identify transcription factor target genes using TargetOrtho2

**DOI:** 10.1016/j.xpro.2025.103680

**Published:** 2025-03-06

**Authors:** Jonathan D. Rumley, Jee Hun Kim, Oliver Hobert

**Affiliations:** 1 Department of Biological Sciences, Columbia University, New York, NY, USA; 2 Howard Hughes Medical Institute, New York, NY 10027, USA

**Keywords:** Bioinformatics, Sequence analysis, Genomics, Model Organisms, Molecular Biology, Gene Expression

## Abstract

TargetOrtho2 uses transcription factor binding site information to predict transcription factor targets in *C. elegans*, based on an *in silico* phylogenetic footprinting approach. Here, we present a protocol to identify transcription factor target genes using a new version of TargetOrtho2. We provide instructions for installing TargetOrtho2 and its required suite of programs, for predicting transcription factor target genes, and for updating and adding new genomes to TargetOrtho2.

## Highlights

• Instructions for local installation of TargetOrtho2 and necessary suite of programs
• Steps for using TargetOrtho2 to predict transcription factor target genes
• Guidance on updating and adding new genomes to TargetOrtho2

## Abstract (editor highlights)

Publisher’s note: Undertaking any experimental protocol requires adherence to local institutional guidelines for laboratory safety and ethics.

## Graphical abstract

**Figure:**
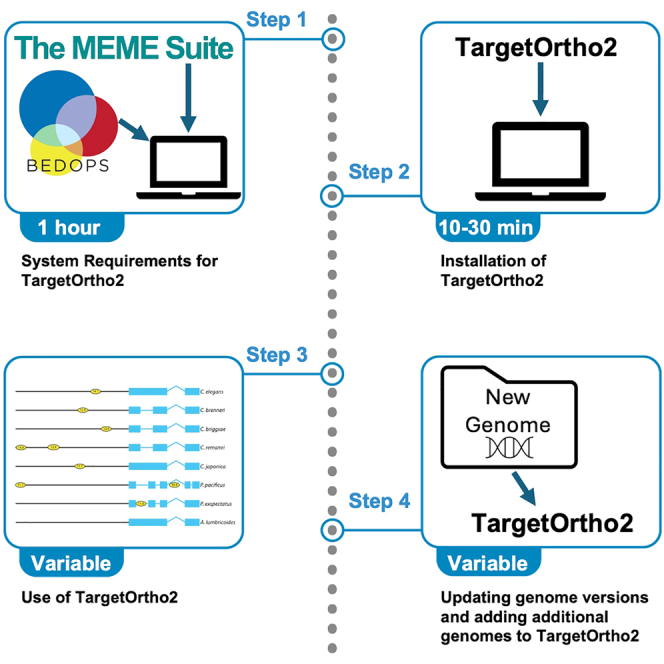

## Before you begin

**Figure 1:**
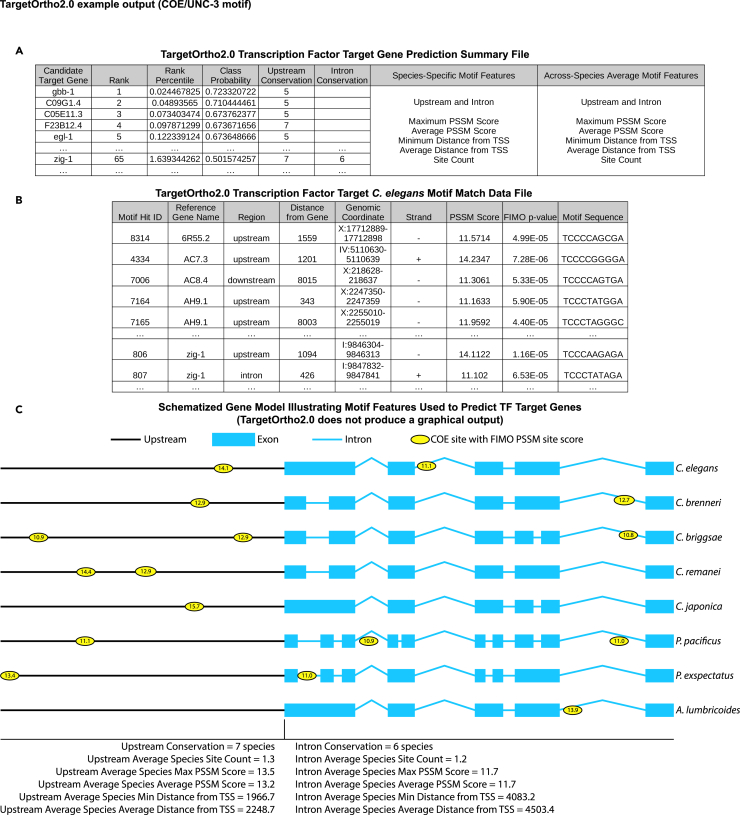
Principle of TargetOrtho2 Shown here is an example output of TargetOrtho2 for the COE motif bound by UNC-3. (A) The main output file is the target gene prediction summary file (ranked_genes_summary.csv), which shows a ranked list of predicted target genes in the reference genome. Genes are ranked by class label probability, which is calculated by a Gaussian process classifier based on the motif features indicated in the table for sites in upstream regions and introns for each gene, as well as the alignment-independent conservation of motifs in upstream regions and introns in orthologous genes. Class label probability ranges from −1 to 1, with higher values indicating greater likelihood to be a target of the transcription factor of interest. (B) TargetOrtho2 also outputs motif match data files for each species with a list of detected motifs assigned to protein-coding genes and the region of each locus in which the site is detected. Other information about each motif is given, as in the table. (C) The bottom portion of this panel illustrates the motif features used to predict transcription factor target genes. Shown is a schematized version of the results for COE sites in the *zig-1* locus. TargetOrtho2 does not produce a graphical output.

We describe here a protocol for the local installation and use of TargetOrtho2. TargetOrtho2 is a program that uses transcription factor binding site information to scan whole genomes for the occurrence of such sites, and associates them with the upstream regions, introns, exons, and downstream regions of genes. Putative target genes are ranked by their likelihood to be true targets based on several transcription factor binding motif features, including, most importantly, phylogenetic conservation of motifs in the upstream regions and introns of orthologous genes (“phylogenetic footprinting”) (Figure 1)^1^^,^^2^ The program, as published, searches the genomes of up to eight nematode species for phylogenetically conserved transcription factor binding sites, and is adaptable to search other well-annotated genomes, such as those of various *Drosophila* species. Several *C. elegans* genetics studies have used or adapted TargetOrtho2 and its predecessor TargetOrtho since its initial publication.^3^^,^^4^^,^^5^^,^^6^^,^^7^^,^^8^^,^^9^^,^^10^^,^^11^ However, since the Galaxy webtool on which previous TargetOrtho versions were running is no longer available, local installations of TargetOrtho2 are necessary. Additionally, since Python 2 is now deprecated, we have converted TargetOrtho2 to Python 3 code.

### System requirements for TargetOrtho2

**Timing: 1 h**

**Figure 2:**
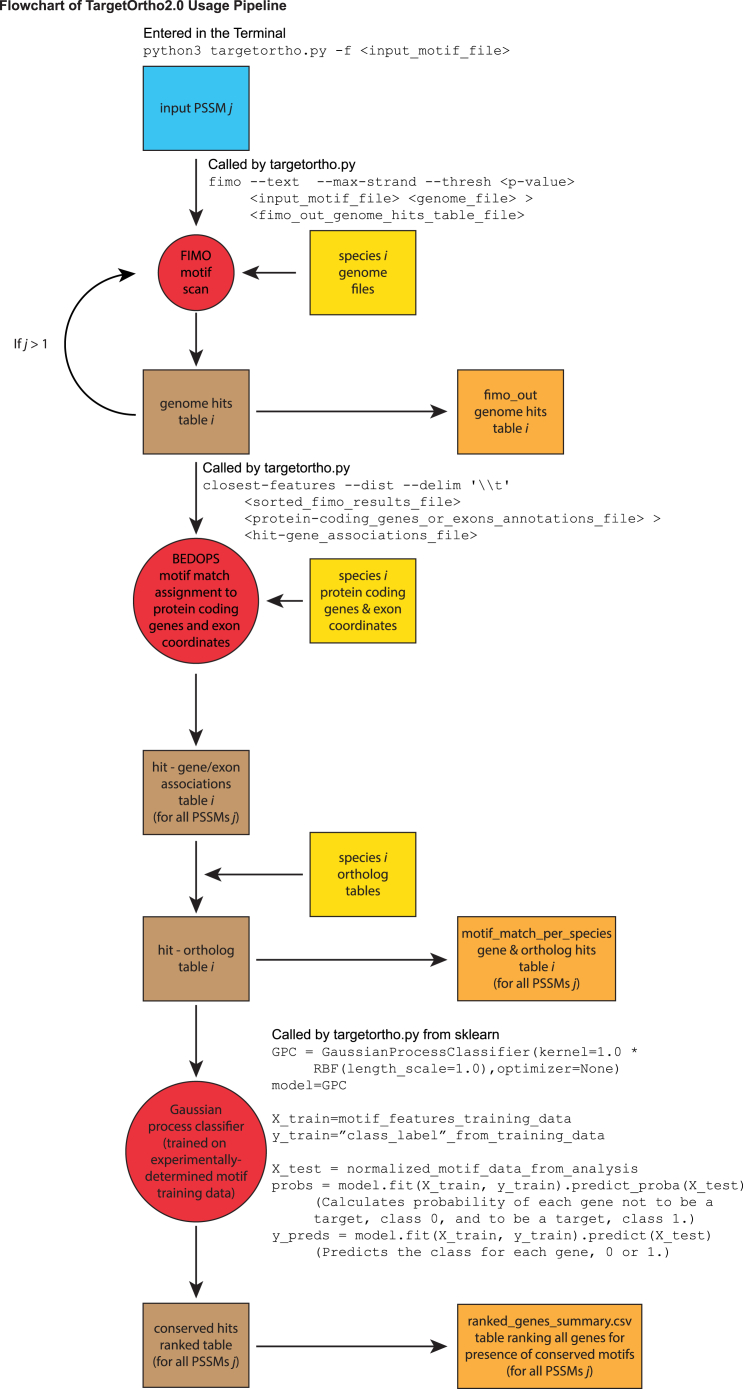
Flowchart showing the pipeline of TargetOrtho2 usage The input is one to five MEME format PSSMs in a text file. The program FIMO from the MEME suite searches all eight nematode genomes for motifs matching the first PSSM, and produces a genome hits table, which is output to the fimo_out folder. If more than one PSSM was in the input file, FIMO searches the genomes for that motif and adds these to the genome hits tables. BEDOPS software (closest-features) matches these genome hits to annotated protein coding genes and upstream regions, introns, exons, and downstream regions for each locus. Orthologous genes from the motif-gene associations tables are matched, and the results are output in the motif_match_per_species folder. A Gaussian process classifier is used to compare normalized motif feature data to motif feature data in a training set to determine which genes are most likely to be true transcription factor targets based on the similarity of their motif features to those of true targets in the training data. Predicted target genes are ranked based on their motif features and their alignment-independent conservation in upstream regions and introns of orthologous genes. The results are outputted in the file <JobID>_ranked_genes_summary.csv. A command used to run targetortho.py and important code for the highlighted major steps are included in the figure.

You can install TargetOrtho2 on macOS, Ubuntu (Linux), and Windows (using Windows Subsystem for Linux—WSL). TargetOrtho2 requires the operating system macOS X version 10.11.6 (El Capitan) or later, Ubuntu 14.04 or later, or Windows 10 or later (with WSL 2). For Ubuntu, ensure that your operating system uses x86_64/AMD architecture. The BEDOPS binary file closest-features requires x86_64/AMD architecture and does not function on systems with ARM-based architecture, such as aarch64. For macOS, TargetOrtho2 also requires Xcode command line tools. TargetOrtho2 also requires Python 3 with the modules sklearn and pandas, the MEME Suite version 4.12.0 or later,^12^^,^^13^ BEDOPS version 2.3.30 or later,^14^ and bedtools version 2.27.1 or later.^15^ A flowchart of TargetOrtho2 usage is provided in Figure 2.

1. System environment installations (Follow the instructions for your operating system).
  a. Windows:
    i. To install WSL 2, open Command Prompt in administrator mode and enter:
      wsl --install
    ii. To see a list of available distributions, in Command Prompt enter:
      wsl --list --online
    iii. To run your desired distribution, in Command Prompt enter:
      wsl --install -d <Distribution_Name>
      (e.g., wsl --install -d Ubuntu-24.04)
      ***Note:*** Your Windows user account can be found in WSL at //mnt/c/Users/<user_name> (e.g. //mnt/c/Users/jonathan).
  b. MacOS:
    i. To install Xcode command line tools, in the Terminal enter:
      xcode-select ––install
      ***Note:*** Make sure this installation is complete before attempting any installations using MacPorts.
2. To install Python3, go to the Python downloads site (https://www.python.org/downloads/).
  a. Download the appropriate installer and follow the installation instructions.
3. To install the sklearn module, enter one of the following commands in the Terminal, and enter the password for your user account:
  a. MacOS:
    sudo pip install scikit-learn
  b. Ubuntu and Windows (WSL):
    sudo apt-get update
    sudo apt-get install python3-sklearn python3-sklearn-lib python-sklearn-doc
4. To install the pandas module, enter one of the following commands in the Terminal:
  a. MacOS:
    sudo pip install pandas
  b. Ubuntu and Windows (WSL):
    sudo apt-get install python3-pandas
5. To install the MEME Suite, go to the MEME Suite download site (https://meme-suite.org/meme/doc/download.html).
  a. Download the latest version of the MEME Suite.
  b. Decompress the downloaded compressed file.
  c. Install the MEME Suite on your computer by following the instructions on the MEME Suite installation site (https://meme-suite.org/meme/doc/install.html?man_type=web).
    ***Note:*** For MacOS we recommend performing the Quick Install using MacPorts. To install MacPorts, go to the MacPorts installation site and follow the instructions for installing MacPorts on your computer’s operating system (https://www.macports.org/install.php).
    ***Note:*** For Ubuntu and Windows (WSL) we recommend performing the Quick Install from Source. Troubleshooting 1.
  d. Make the MEME Suite program fimo executable by copying it to the directory /usr/local/bin.^16^
    i. Following installation, find fimo in the directory meme-<version>/src (e.g., meme-5.5.7/src).
    ii. In MacOS, following the installation by MacPorts, also find fimo in the directory /opt/local/bin.
    iii. To confirm the location of fimo, navigate to one of the above-mentioned directories, and enter the following command in the Terminal:
      ls
        The file fimo should appear in the output.
    iv. Copy fimo to /usr/local/bin by entering the following command in the Terminal:
      sudo cp fimo /usr/local/bin
6. To install BEDOPS, go to the BEDOPS download site (https://bedops.readthedocs.io/en/latest/index.html).
  a. MacOS:
    i. Download the installer package for OS X, and follow the instructions.
      ***Note:*** This should install all the BEDOPS binary files in the directory /usr/local/bin.
  b. Ubuntu and Windows (WSL):
    i. Download the BEDOPS binary files for Linux.
    ii. Extract the compressed file by navigating to the Downloads directory and entering the following command in the Terminal:
      sudo tar jxvf bedops_linux_x86_64-v<verson>.tar.bz2
      (e.g., sudotar jxvf bedops_linux_x86_64-v2.4.41.tar.bz2)
    iii. Copy the BEDOPS binary files to /usr/local/bin by entering the following command in the Terminal:
      sudo cp bin/∗ /usr/local/bin
7. To install bedtools, enter the appropriate command in the Terminal:
  a. MacOS:
    sudo port install bedtools
  b. Ubuntu and Windows (WSL):
    sudo apt-get install bedtools

## Key resources table

**Table:** 

REAGENT or RESOURCE	SOURCE	IDENTIFIER
**Software and algorithms**		

macOS X version 10.11.6 (El Capitan) or later	Apple	https://www.apple.com/app-store/
Ubuntu 14.04 or later	Ubuntu	https://ubuntu.com/
Windows 10 or later	Microsoft	https://www.microsoft.com/en-us/software-download/
Windows Subsystem for Linux (WSL)	Microsoft	https://learn.microsoft.com/en-us/windows/wsl/install
Xcode command line tools	Xcode	N/A
MEME Suite version 4.12.0 or later	Bailey et al.^12^^,^^13^	https://meme-suite.org/meme/doc/download.html https://meme-suite.org/meme/doc/install.html?man_type=web
fimo	Grant et al.^16^	https://meme-suite.org/meme/doc/download.html https://meme-suite.org/meme/doc/install.html?man_type=web
BEDOPS version 2.3.30 or later	Neph et al.^14^	https://bedops.readthedocs.io/en/latest/
bedtools version 2.27.1 or later	Quinlan et al.^15^	https://bedtools.readthedocs.io/en/latest/content/installation.html
Python 3	Python	https://www.python.org/downloads/
sklearn	Python	https://pypi.org/project/scikit-learn/0.20.4/
pandas	Python	https://pandas.pydata.org/pandas-docs/version/0.24/install.html
TargetOrtho2	Glenwinkel et al.^1^	https://github.com/loriglenwinkel/TargetOrtho2.0
TargetOrtho2_Python3	This manuscript (https://doi.org/10.5281/zenodo.14750829)	https://github.com/jdrumley1989/TargetOrtho2_Python3
execute_copy_terminal.py	This manuscript	https://github.com/jdrumley1989/TargetOrtho2_Python3
matrix2meme	Bailey et al.^12^^,^^13^	https://meme-suite.org/meme/doc/download.html https://meme-suite.org/meme/doc/install.html?man_type=web
TargetOrtho_motif_match_motif_search_terminal.R	This manuscript	https://github.com/jdrumley1989/TargetOrtho2_Python3
TargetOrthoFIMO_motif_search_terminal.R	This manuscript	https://github.com/jdrumley1989/TargetOrtho2_Python3
R	R-project	https://cran.r-project.org/bin/macosx/
WormBase ParaSite	Howe et al.,^17^ Howe et al.,^18^ Sternberg et al.,^19^ Yoshida et al.,^20^ and Coghlan et al.^21^	https://ftp.ebi.ac.uk/pub/databases/wormbase/parasite/releases/, https://parasite.wormbase.org/ftp.html
WormBase ParaSite BioMart	Howe et al.,^17^ Howe et al.,^18^ Sternberg et al.,^19^ Yoshida et al.,^20^ and Coghlan et al.^21^	https://parasite.wormbase.org/biomart/martview/ef512a6e58b0958918621c301545f291
make_training_data.py	This manuscript	https://github.com/jdrumley1989/TargetOrtho2_Python3

## Step-by-step method details

### Installation of TargetOrtho2

**Timing: 10–30 min**

This step instructs how to install TargetOrtho2 on a local computer.

1. Download TargetOrtho2 as a compressed (.zip) file from the TargetOrtho2_Python3 GitHub repository (https://github.com/jdrumley1989/TargetOrtho2_Python3).
  a. In the upper right corner, click the green “Code” button.
  b. Select “Download ZIP” to download the compressed file called TargetOrtho2_Python3-main.zip.
2. Decompress the file.
3. Open the directory TargetOrtho2_Python3-main.
4. In this directory, decompress the file TargetOrtho2_Python3_github.zip.
5. In the Terminal, navigate to the directory Downloads/TargetOrtho2_Python3-main/TargetOrtho2_Python3_github.
6. Run the file setup.command by entering the following command in the Terminal:

python3 setup.command

***Note:*** This downloads and decompresses the genome sequence files that TargetOrtho2 searches to identify transcription factor binding site motifs. These genome sequence files are for *Caenorhabditis elegans*, *Caenorhabditis briggsae*, *Caenorhabditis brenneri*, *Caenorhabditis remanei*, *Caenorhabditis japonica*, *Pristionchus pacificus*, *Pristionchus exspectatus*, and *Ascaris lumbricoides*. These genome sequence files are deposited in the genomes directory. Troubleshooting 2.

### Use of TargetOrtho2

**Timing: Variable**

This step instructs how to run TargetOrtho2 to predict transcription factor binding sites and transcription factor target genes in the *C. elegans* or *P. pacificus* genomes.

7. To run TargetOrtho2, in the Terminal, navigate to the directory TargetOrtho2_Python3_github.
8. Run the script targetortho.py by entering a command such as the following in the Terminal troubleshooting 3:
  python3 targetortho.py -f <input_file>
  (e.g., python3 targetortho.py -f data/input_motif_examples/COE_motif_PSSM_meme4.txt)
  ***Note:*** Entering this command will run targetortho.py using the indicated input file, the default maximum fimo p-value of 0.0001, and the default reference species of *C. elegans*. Troubleshooting 4.
  a. Input files for TargetOrtho2 are Position Specific Scoring Matrices (PSSMs) in MEME format (Figure 3).
    i. TargetOrtho2 accepts either log-odds matrices or letter-probability matrices. Troubleshooting 5.
    ii. If your PSSM is in Cis-BP format (or another format), it must be converted to MEME format. To convert all PSSMs in a specified directory from Cis-BP format to MEME format, use the script execute_copy_terminal.py included in the GitHub download. To run execute_copy_terminal.py, enter the following command in the Terminal:
      python3 execute_copy_terminal.py <input_folder> <output_folder>
      (e.g., python3 execute_copy_terminal.py cis-bp_format_input_folder meme_formate_output_folder)
      ***Note:*** Below see an example of the conversion of a Cis-BP format PSSM to MEME format:

**Figure:**
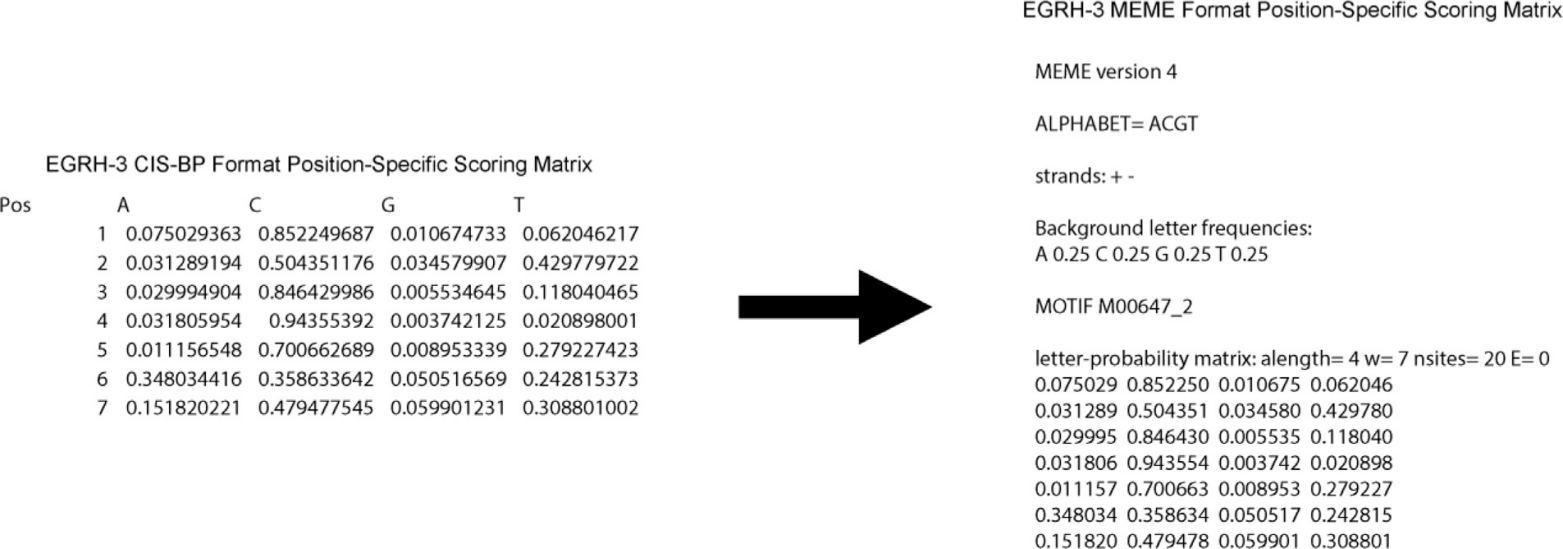
    iii. To convert individual PSSMs from a raw matrix to MEME format, use the script matrix2meme included in the MEME suite. To use matrix2meme, copy it to the directory /usr/local/bin to make it executable. From the Downloads directory, enter the following command in the Terminal:
      sudo cp meme-<version>/scripts/matrix2meme /usr/local/bin
      (e.g., sudo cp meme-5.5.7/scripts/matrix2meme /usr/local/bin)
      To run matrix2meme, enter the following command in the Terminal:
      matrix2meme < <input_file> >> <output_file>
      (e.g., matrix2meme < raw_matrix_PSSM_input.txt >> MEME_matrix_PSSM_output.txt)
      ***Note:*** Below see an example of the conversion of a raw PSSM to MEME format:

**Figure:**
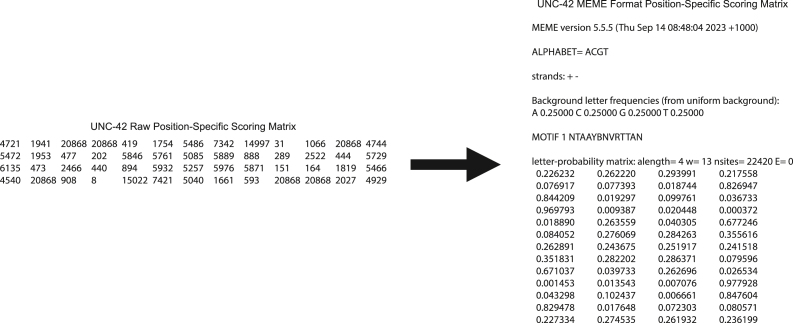

**Figure 3:**
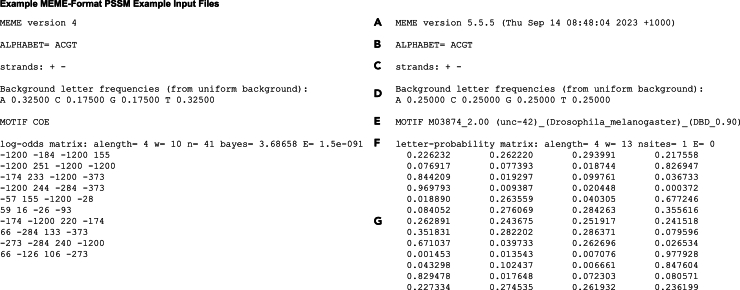
Examples of MEME format PSSMs These PSSMs should be .txt files. The sections of the PSSMs are as follows: (A) The MEME version from which the PSSM file was produced. This does not have to be accurate for the proper functioning of TargetOrtho2. (B) The alphabet used for the PSSM. For DNA this is “ACGT”. (C) The strands in which to search for motifs. This should be “+ -” for searching both + and - DNA strands. (D) The background frequency of each DNA base to expect in the reference genome. (E) The name of the motif. (F) Information about the PSSM, most importantly the type of matrix (log-odds or letter-probability), the number of classes of letters to expect (alength; 4 for DNA), and the number of letters to expect in the motif (w). (G) The PSSM with four columns (one for each DNA base in alphabetical order; i.e., ACGT) and the same number of rows as the number of bases in the motif, with each row representing successive positions in the motif. The values in each row are representations of the probabilities of each of the bases appearing at each position.^1^^,^^22^
9. Use the following options to customize the targetortho.py run:

-p <maximum_p-value> (e.g. -p 0.0001)

-d <maximum_distance_from_gene> (e.g. -d 500)

-o <output_directory> (e.g. -o my_output)

-j <job_ID> (e.g. -j job1)

-s <species_file> (e.g. -s all_species.txt)

-r <reference_species> (*C. elegans* or *P. pacificus*; e.g. -r C.elegans)

***Note:*** By default, the output directory is titled <JobID>_TargetOrtho2.0_Results.

***Note:*** The species file is a text file with a list of nematode species the genomes of which are searched for transcription factor binding sites and used to rate the likelihood of transcription factor target genes. By default, these species are *Caenorhabditis elegans*, *Caenorhabditis briggsae*, *Caenorhabditis brenneri*, *Caenorhabditis remanei*, *Caenorhabditis japonica*, *Pristionchus pacificus*, *Pristionchus exspectatus*, and *Ascaris lumbricoides*. Including a species file allows only a subset of these species’ genomes to be used in the analysis. In the species file, species names must be abbreviated and each species must be listed in an individual line as follows:

c_eleg

c_brig

c_bren

c_japo

c_rema

p_paci

p_exsp

a_lumb

***Note:*** Transcription factor target gene prediction is only performed if five or eight species’ genomes are searched. If five species are included in the list, training data from *Caenorhabditis elegans*, *Caenorhabditis briggsae*, *Caenorhabditis brenneri*, *Caenorhabditis remanei*, and *Caenorhabditis japonica* are used to make target gene predictions. If eight species are included, all eight above species’ training data are used.

### Updating genome versions and adding additional genomes to TargetOrtho2

**Timing: Variable**

This step instructs how to update genome versions and add additional genomes to TargetOrtho2.

10. Download the new genome files from WormBase ParaSite (https://ftp.ebi.ac.uk/pub/databases/wormbase/parasite/releases/, https://parasite.wormbase.org/ftp.html) or from an alternative source.
  a. Place the genome files in the genomes directory.
  b. Ensure the file names are in the same format as the existing genome files.
11. Download the relevant ortholog files from the WormBase ParaSite BioMart (https://parasite.wormbase.org/biomart/martview/ef512a6e58b0958918621c301545f291).
  a. In the Query Filters tab, under the heading SPECIES, check Genome, and select Caenorhabditis elegans (PRJNA13758) to use *C. elegans* as the reference species.
  b. In the Output Attributes tab, under the heading GENE, check Gene name, and under the heading ORTHOLOGUES, check gene name and Homology type for one of the desired species. (e.g., For *C. briggsae*, under Caenorhabditis briggsae (PRJNA10731) Orthologues, select Caenorhabditis briggsae (PRJNA10731) gene name and Homology type).
    i. If gene names do not exist for a particular species, check gene stable ID instead of gene name.
      **CRITICAL:** Ensure the selection is made in this order, so that the output file is properly formatted.
  c. Click the Results button in the upper left corner and preview the output orthologs file.
    i. If the gene names for the species are not displayed, then click on the Output Attributes tab again, deselect gene name and Homology type, and check gene stable ID and Homology type. Click the Results button again.
  d. Download the output orthologs file as a TSV file.
    i. Under Export all results to, select File and TSV and click the Go button.
  e. Rename the downloaded file identically to the existing corresponding orthologs file.
    i. (e.g., c_eleg_c_brig_ortho.txt).
  f. Place the file in the ortholog_files directory.
  g. Repeat for each species for which you want to update or add a genome.
    ***Note:*** If you use ortholog data from another source, ensure that the data are compiled and that the file is named in the same format as existing orthologs files.
12. Download the relevant exon annotations files from the WormBase ParaSite BioMart.
  a. In the Query Filters tab, under the heading SPECIES, check Genome, and select the species of interest (e.g., Caenorhabditis elegans (PRJNA13758)).
  b. In the Output Attributes tab, under GENE check Chromosome/scaffold name, under EXONS check Exon region start (bp) and Exon region end (bp), and under GENE check Gene stable ID, Gene name, and Strand.
    **CRITICAL:** It is necessary to make the selection in this order for the output file to be formatted properly.
  c. Click the Results button in the upper left corner and view the preview of the file.
  d. Download the exon annotations file as a TSV file.
    i. Under Export all results to, select File and TSV and click the Go button.
  e. Rename the downloaded file in the same format as the existing corresponding exon annotations file.
    i. (e.g., For the WS290 version of the *C. elegans* exon annotations, name the file mart_export_c_eleg_exonsWS290.txt).
  f. If gene names are not present for a particular species, copy and paste the Gene stable ID column to the Gene name column.
  g. Make a copy of the file and delete the headers from the copied file.
    i. Rename this file with “_no_header” added to the end of the original file’s name. (e.g., mart_export_c_eleg_exonsWS290_no_header.txt).
  h. Sort the file using bedtools by entering the following command in the Terminal from the directory in which the exon annotations file is present:
    bedtools sort -i <no_header_input_file> > <output_bed_file>
    (e.g., bedtools sort -i mart_export_c_eleg_exonsWS290_no_header.txt > mart_export_c_eleg_exonsWS290.bed)
  i. Move the sorted exon annotations file to the gene_coords directory.
  j. Repeat for each species.
    ***Note:*** If you use exon annotations data from another source, ensure that the data are compiled and that the file is named in the same format as existing exon annotations files.
13. Download the relevant protein coding gene annotations files from the WormBase ParaSite BioMart.
  a. In the Query Filters tab, under the heading SPECIES, check Genome, and select the species of interest (e.g., Caenorhabditis elegans (PRJNA13758)).
  b. In the Output Attributes tab, under GENE check Chromosome/scaffold name, Gene start (bp), Gene end (bp), Gene stable ID, Gene name, and Strand.
    **CRITICAL:** It is necessary to make the selection in this order for the output file to be formatted properly.
  c. Click the Results button in the upper left corner and view the preview of the file.
  d. Download the protein coding gene annotations file as a TSV file.
    i. Under Export all results to, select File and TSV and click the Go button.
  e. Rename the downloaded file in the same format as the existing corresponding protein coding gene annotations file.
    i. (e.g., For the WS290 version of the *C. elegans* protein coding gene annotations, name the file mart_export_c_eleg_protein_coding_genesWS290.txt).
  f. If gene names are not present for a particular species, copy and paste the Gene stable ID column to the Gene name column.
  g. Make a copy of the file and delete the headers from the copied file.
    i. Rename this file with “_no_header” added to the end of the original file’s name. (e.g., mart_export_c_eleg_protein_coding_genesWS290_no_header.txt).
  h. Sort the file using bedtools by entering the following command in the Terminal from the directory in which the protein coding genes annotations file is present:
    bedtools sort -i <no_header_input_file> > <output_bed_file>
    (e.g., bedtools sort -i mart_export_c_eleg_protein_coding_genesWS290_no_header.txt > mart_export_c_eleg_protein_coding_genesWS290.bed)
  i. Move the sorted protein coding gene annotations file to the gene_coords directory.
  j. Repeat for each species.
    ***Note:*** If you use protein coding genes annotations data from another source, ensure that the data are compiled and that the file is named in the same format as existing protein coding genes annotations files.
14. Edit the python script targetortho.py.
  a. Open the file targetortho.py in a text editor or integrated development environment (IDE).
  b. Make a copy of the file.
  c. Update or add the genome file names in the genomesDic section starting with line 99.
    i. These must match the genome file names in the genomes directory.
    ii. (e.g., For the WBPS19 version of the *C. elegans* genome:
      "c_eleg":"caenorhabditis_elegans.PRJNA13758.WBPS19.genomic.fa")
  d. Update or add the annotations versions in the versionDic section starting with line 107.
    i. These must match the end of the exon annotations and the protein coding genes annotations file names.
    ii. (e.g., For mart_export_c_eleg_protein_coding_genesWS290.bed, the corresponding line in targetortho.py should read as follows:
      "c_eleg":"WS290")
  e. If adding additional genomes to the 8 included in the download, edit the following line to predict target genes using more than 8 genomes:
    if len(speciesList)==8: (line 923)
    i. Change this line to the following:
      if len(speciesList)>=8:
      ***Note:*** This will allow TargetOrtho2 to make target gene predictions using the binding motif data from all included genomes, but the training data, upon which the predictions are based, only include motif data from the 8 species included in TargetOrtho2.
15. Write a text file with a list of all species to be used for the analysis, using abbreviated names, as in the example below:

c_eleg

c_brig

c_bren

c_japo

c_rema

p_paci

p_exsp

a_lumb

c_trop

16. To run TargetOrtho2 with the updated and/or added genomes, enter a command into the Terminal such as the following:

python3 targetortho_mod.py -f <input_file> -s <species_file>

(e.g., python3 targetortho_mod.py -f data/input_motif_examples/COE_motif_PSSM_meme4.txt -s all_species.txt)

17. Make an updated training data file using updated and/or added genomes and annotations.
  a. Run TargetOrtho2 on each of the included PSSMs in the data/input_motif_examples directory.
    i. For COE_motif_PSSM_meme4.txt, use a p-value threshold of 0.00075.
       (e.g., python3 targetortho_mod.py -f data/input_motif_examples/COE_motif_PSSM_meme4.txt -s all_species.txt -p 0.00075).
    ii. For ASE_motif_PSSM_meme4.txt, use a p-value threshold of 0.00025.
    iii. For AIY_motif_PSSM_meme4.txt, use a p-value threshold of 0.00026.
  b. Copy the <job_id>_all_info_normed.txt file from the output of each of these runs of TargetOrtho2 to the data/training_data directory.
  c. Use a text editor or an IDE to open the python script make_training_data.py in the data/training_data directory.
  d. Edit make_training_data.py
    i. Replace the existing names for the COE, ASE, and AIY motif <job_id>_all_info_normed.txt files with those you have produced.
    ii. If desired, change the file name in output_path to your desired file name.
  e. Run make_training_data.py by entering the following command in the Terminal from the data/training_data directory:
    python3 make_training_data.py
    ***Note:*** The output file is the new training data file that includes motif data from the updated and/or added genomes.
  f. If you add new species genomes, edit your modified version of targetortho.py (e.g., targetortho_mod.py) in a text editor or IDE.
    i. Change the line that was edited in step 14e to its original form. (e.g., Change
      if len(speciesList)>=8:
      back to
      if len(speciesList)==8:
    ii. Below the line that follows this one, insert the following lines of code to analyze 9 genomes:
      If len(speciesList)==9:
       train = pd.read_csv("%s/data/training_data/training_data_9species.txt" %(TargetOrtho_path,sep=’∖t’)
      ***Note:*** Adjust the code according to the number of genomes you intend to analyze and the name of the training data file.
  g. Run your modified version of targetortho.py by entering a command such as the following in the Terminal from the TargetOrtho2_Python3_github directory:
    python3 targetortho_mod.py -f <input_file> -s <species_file>
    (e.g. python3 targetortho_mod.py -f data/input_motif_examples/COE_motif_PSSM_meme4.txt -s all_species.txt)

## Expected outcomes

All output files are in the output directory <JobID>_TargetOrtho2.0_Results. The output files are as follow and contain the data described below.

<JobID>_input_summary.txt contains information about the input of the TargetOrtho2 run, including the job ID, the file path for the motif file, the list of species used in the analysis, the p-value threshold used in the fimo analysis, the maximum distance from the TSS searched for motif matches, and the command entered in the Terminal to run the analysis.

The files fimo_out/<JobID>_c_eleg_fimo.txt, <JobID>_c_brig_fimo.txt, <JobID>_c_bren_fimo.txt, <JobID>_c_japo_fimo.txt, <JobID>_c_rema_fimo.txt, <JobID>_p_paci_fimo.txt, <JobID>_p_exsp_fimo.txt, and <JobID>_a_lumb_fimo.txt contain the results of the fimo analysis for each species. These files contain information on all the sites detected in each species’ genome associated with their genomic locations. The quality of each site is indicated by a PSSM score and a p-value, based on how well each site conforms to the consensus sequence of the binding site. These files are produced by fimo when fimo is called by targetortho.py using the following line of code (Figure 2):

fimo --text --max-strand --thresh <p-value> <input_motif_file> <genome_file> > <fimo_out_genome_hits_table_file>

(e.g., fimo --text --max-strand --thresh 0.0001 data/input_motif_examples/COE_motif_PSSM_meme4.txt genomes/caenorhabditis_elegans.PRJNA13758.WBPS10.genomic.fa > j202516162038_TargetOrtho2.0_Results/fimo_out/c_eleg_fimo.txt)

These files are formatted as below, where sequence_name is the chromosome or scaffold in which the motif is located:

**Figure:**
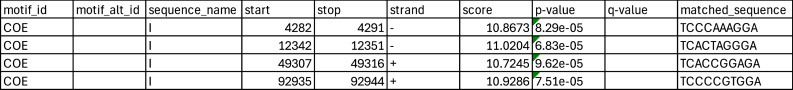

The files motif_match_data_per_species/<JobID>_c_eleg_genome_motif_match_results.csv, <JobID>_c_brig_genome_motif_match_results.csv, <JobID>_c_bren_genome_motif_match_results.csv, <JobID>_c_japo_genome_motif_match_results.csv, <JobID>_c_rema_genome_motif_match_results.csv, <JobID>_p_paci_genome_motif_match_results.csv, <JobID>_p_exsp_genome_motif_match_results.csv, and <JobID>_a_lumb_genome_motif_match_results.csv contain the motif results for each species with sites associated with the genes in the loci of which they are present and with their positions within the loci (i.e. upstream, intron, exon, and downstream). The quality of each site is indicated by a PSSM score and a p-value, based on how well each site conforms to the consensus sequence of the binding site. (Figure 1B). These files are produced by several parts of the code. In particular, gene and exon associations are produced by the BEDOPS program closest-features, which is called by targetortho.py using the following line of code (Figure 2):

closest-features --dist --delim '∖∖t' <sorted_fimo_results_file> <protein-coding_genes_or_exons_annotations_file> > <hit-gene_associations_file>

(e.g. closest-features --dist --delim '∖∖t' j202462817501_TargetOrtho2.0_Results/fimo_out/j202462817501_c_eleg.bed.sorted gene_coords/mart_export_c_eleg_protein_coding_genesWS258.bed > temp_files/j202462817501/c_eleg_associated_genes.bed;

closest-features --dist --delim '∖∖t' j202462817501_TargetOrtho2.0_Results/fimo_out/j202462817501_c_eleg.bed.sorted gene_coords/mart_export_c_eleg_exonsWS258.bed > temp_files/j202462817501/c_eleg_associated_exons.bed)

These files are formatted as below:

**Figure:**
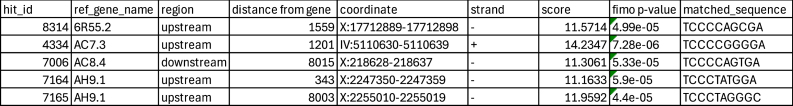

<JobID>_all_info_normed.txt contains motif features of all the genes identified to have sites that match the input motif. These data are normalized such that the reported values are calculated using the following formula:

normalizedvalue=(value−columnminimum)÷(columnmaximum−columnminimum)

Motif features for each gene included in these data are upstream and intron conservation (calculated by dividing the number of species in which the site is conserved by the total number of species in the analysis), the average maximum PSSM score for all species, the average mean PSSM score for all species, the average minimum distance from the TSS for all species, the average mean distance from the TSS for all species, the average site count for all species, and each of these values for each species. These data are used by the Gaussian Process Classifier to compare motif features to the training data to predict target genes of the transcription factor of interest. This file is produced by merging the motif feature data for upstream and intronic motifs and normalizing the data. The final <JobID>_all_info_normed.txt file is produced by the following function:

norm_data(df,min_scores,max_scores,min_dist,max_dist,max_count)

This file is formatted as below:

**Figure:**
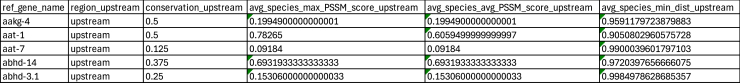

**Figure:**
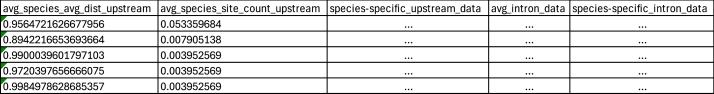

<JobID>_TargetOrtho2_ranked_genes_summary.csv contains a ranked list of genes with sites matching the input motif in the reference species. These genes are ranked based on how well their motif features conform to those in the training data for true targets, which is quantified by the value classifier label probability (class_prob). Classifier label probability has values from −1 to 1, with higher values indicating greater probability of a gene being a true target. Genes with classifier label probability values of 0 to 1 are predicted to be true targets. Genes with classifier label probability values of −1 to 0 are predicted not to be true targets. Genes are ranked in order of their classifier label probability, such that the gene with the highest classifier label probability is ranked first, and subsequent ranks are assigned to genes with successively lesser classifier label probabilities in descending order. This file also contains the non-normalized motif feature data for each gene. These data are the same as those in the file <JobID>_all_info_normed.txt. (Figure 1A). Target gene predictions are made using the Gaussian Process Classifier from sklearn, which makes target gene predictions by comparing the normalized motif feature data to the training data. The Gaussian Process Classifier is called by targetortho.py using the following lines of code (Figure 2):

GPC = GaussianProcessClassifier(kernel=1.0 ∗ RBF(length_scale=1.0),optimizer=None)

model=GPC

X_train=motif_features_training_data

y_train="class_label"_from_training_data

X_test = normalized_motif_data_from_analysis

probs = model.fit(X_train, y_train).predict_proba(X_test)

y_preds = model.fit(X_train, y_train).predict(X_test)

(i.e. GPC = GaussianProcessClassifier(kernel=1.0 ∗ RBF(length_scale=1.0),optimizer=None)

model=GPC

X_train= train[feature_set]

y_train= train["class_label"]

X_test = df_normed[feature_set]

probs = model.fit(X_train, y_train).predict_proba(X_test)

y_preds = model.fit(X_train, y_train).predict(X_test))

This file is formatted as below:

**Figure:**
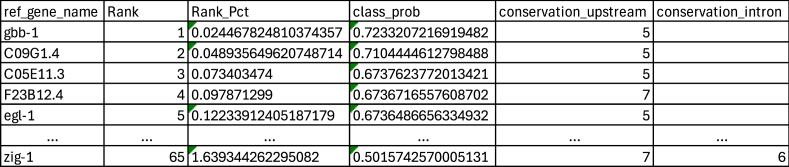

**Figure:**
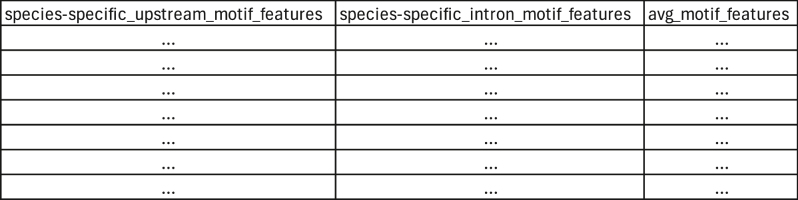

The main applications of these data are to predict binding sites for transcription factors in the loci of genes of interest and to predict target genes of transcription factors of interest.

To predict transcription factor binding sites in the loci of genes of interest, the most relevant output files are those in the directory motif_match_data_per_species, and secondarily the files in the directory fimo_out. The files in the directory motif_match_data_per_species can be searched for genes of interest to identify predicted transcription factor binding sites in the upstream, intronic, exonic, or downstream regions of genes of interest in the genomes of any of the eight species used in the analysis. The files in the directory fimo_out can be searched for genomic regions of interest to identify predicted transcription factor binding sites. These files are primarily useful if an error prevents the program from producing the files in the directory motif_match_data_per_species. To determine which genes are associated with predicted transcription factor binding sites using these data, it is necessary to manually associate the genomic regions with the genes of interest.

Most of these files can be opened using Microsoft Excel or another spreadsheet program. For large output files, however, which can be generated if the *p*-value threshold is set high (weakly stringent), one can filter for the sites of interest based on either the associated gene name (for files in the motif_match_data_per_species directory) or the genomic coordinates of interest (for files in the fimo_out directory) using the R scripts TargetOrtho_motif_match_motif_search_terminal.R or TargetOrthoFIMO_motif_search_terminal.R, respectively. To run these scripts, enter the following commands from the directory TargetOrtho2_Python3_github in the Terminal, respectively.

Rscript TargetOrtho_motif_match_motif_search_terminal.R <JobID>_TargetOrtho2.0_Results/motif_match_data_per_species/<input_file> <reference_gene>

(e.g. Rscript TargetOrtho_motif_match_motif_search_terminal.R j2025160324_TargetOrtho2.0_Results/motif_match_data_per_species/j2025160324_c_eleg_genome_motif_match_results.csv zig-1)

Rscript TargetOrthoFIMO_motif_search_terminal.R <JobID>_TargetOrtho2.0_Results/fimo_out/<input_file> <chromosome/contig/scaffold> <start_search_position> <stop_search_position>

(e.g. Rscript TargetOrthoFIMO_motif_search_terminal.R j2025160324_TargetOrtho2.0_Results/fimo_out/ j2025160324_c_eleg_fimo.txt I 9800000 9900000)

To identify probable target genes of a transcription factor of interest, the most relevant output file is <JobID>_TargetOrtho2_ranked_genes_summary.csv. As noted above, this file ranks genes based on classifier label probability (class_prob). Classifier label probabilities of 0–1 indicate that a gene is increasingly likely to be a true target of the transcription factor of interest, and classifier label probabilities of 0 to −1 indicate that a gene is increasingly unlikely to be a true target. One way that these data can be used is to determine if the top set of predicted target genes (e.g., top ten predicted target genes) are predicted to be true targets of the transcription factor of interest. For instance, for the transcription factor UNC-3, which binds to the COE motif, do the top ten predicted targets have a classifier label probability greater than 0 when the *p*-value threshold is set to 0.0001 and there is no maximum distance limit from the TSS for the motif search?

**Table:** 

ref_gene_name	Rank	Rank_Pct	class_prob
gbb-1	1	0.024467824810374357	0.7233207216919482
C09G1.4	2	0.048935649620748714	0.7104444612798488
C05E11.3	3	0.07340347	0.6737623772013421
F23B12.4	4	0.0978713	0.6736716557608702
egl-1	5	0.12233912405187179	0.6736486656334932
R02D5.4	6	0.14680694886224616	0.6723427603446339
ace-2	7	0.1712747736726205	0.6712968907875734
mcu-1	8	0.19574259848299486	0.6646822610653658
Y37E11AL.6	9	0.2202104232933692	0.6631672224920635
F32B5.2	10	0.24467824810374358	0.6586070570857601

The data in the above table show that all the top ten predicted target genes for UNC-3 using the above specified settings are predicted to be true targets, since they all have classifier label probabilities greater than 0.

## Limitations

As noted in Mercatelli et al., 2020,^23^ TargetOrtho2 functions best in identifying transcription factor target genes in compact genomes, such as those of nematodes. Since it predicts transcription factor targets using motifs that are within potential target genes’ loci (either upstream or in introns), TargetOrtho2 would be expected to perform more poorly if used to predict transcription factor target genes in organisms with highly distal enhancers outside of genes’ loci.

## Troubleshooting

### Problem 1

For Ubuntu and Windows (WSL**)**, you may have trouble installing MEME Suite from source (This refers to step 5c in the section system requirements for TargetOrtho2).

### Potential solution

• If any commands fail because you do not have permission to perform an action, enter sudo before the command as follows, and enter your user account password, if asked:

sudo <command>

(e.g. sudo make)

• Delete the meme-<version> directory by entering the following command in the Terminal from the Downloads directory:

rm -r meme-<version>

(e.g. rm -r meme-5.5.7)

• Install development tools by entering the following command in the Terminal:

sudo apt install build-essential

• Install zlib by entering the following command in the Terminal:

sudo apt-get install zlib1g-dev

• Install assorted common utilities by entering the following commands in the Terminal:

sudo apt-get install autoconf

sudo apt-get install automake

sudo apt-get install libtool

• Decompress the MEME Suite compressed file again and follow the installation instructions.
• Follow the troubleshooting instructions on the MEME Suite installation site for additional help.

### Problem 2

The script setup.command may fail. We have noted this particularly in Ubuntu. (This refers to step 6 in the section installation of TargetOrtho2).

### Potential solution

• Manually download the genome files (ending in genomic.fa.gz) from the following URLs:

 *C. elegans*: https://ftp.ebi.ac.uk/pub/databases/wormbase/parasite/releases/WBPS10/species/caenorhabditis_elegans/PRJNA13758/

 *C. briggsae*: https://ftp.ebi.ac.uk/pub/databases/wormbase/parasite/releases/WBPS10/species/caenorhabditis_briggsae/PRJNA10731/

 *C. brenneri*: https://ftp.ebi.ac.uk/pub/databases/wormbase/parasite/releases/WBPS10/species/caenorhabditis_brenneri/PRJNA20035/

 *C. remanei*: https://ftp.ebi.ac.uk/pub/databases/wormbase/parasite/releases/WBPS10/species/caenorhabditis_remanei/PRJNA53967/

 *C. japonica*: https://ftp.ebi.ac.uk/pub/databases/wormbase/parasite/releases/WBPS10/species/caenorhabditis_japonica/PRJNA12591/

 *P. pacificus*: https://ftp.ebi.ac.uk/pub/databases/wormbase/parasite/releases/WBPS10/species/pristionchus_pacificus/PRJNA12644/

 *P. exspectatus*: https://ftp.ebi.ac.uk/pub/databases/wormbase/parasite/releases/WBPS10/species/pristionchus_exspectatus/PRJEB6009/

 *A. lumbricoides*: https://ftp.ebi.ac.uk/pub/databases/wormbase/parasite/releases/WBPS10/species/ascaris_lumbricoides/PRJEB4950/

• Transfer these files to the genomes folder in the TargetOrtho2_Python3_github folder.
• In the Terminal, navigate to the directory TargetOrtho2_Python3_github.
• Decompress the genome files by entering the following command:

gunzip -r genomes

### Problem 3

When targetortho.py encounters an error, it deletes all the output files. You may need to troubleshoot errors in running targetortho.py. (This is related to step 8 in the section use of TargetOrtho2).

### Potential solution

• To prevent targetortho.py from deleting output files when it encounters an error, comment out the following code in line 1033 in targetortho.py:

clear_error()

(i.e. #clear_error())

### Problem 4

When attempting to run targetortho.py, you may receive an error message indicating that a necessary Python module, such as scipy, is not installed. (This refers to step 8 in the section use of TargetOrtho2).

### Potential solution

• Install the indicated module.
• To install scipy, enter oneEeeeee of the following commands in the Terminal:

MacOS:

sudo pip install scipy

Ubuntu and Windows (WSL):

sudo apt-get install python3-scipy

### Problem 5

Some input PSSMs result in empty fimo output files. This is due to the *p*-value threshold being set too low (stringent) for the particular input motif. (This refers to step 8ai in the section use of TargetOrtho2).

### Potential solution

• To resolve this error, set the p-value threshold higher.

## Resource availability

### Lead contact

Further information and requests for resources and reagents should be directed to and will be fulfilled by the lead contact, Oliver Hobert (or38@columbia.edu).

### Technical contact

Technical questions on executing this protocol should be directed to and will be answered by the technical contacts, Jonathan D. Rumley (jdr2203@columbia.edu) and Jee Hun Kim (jk4213@columbia.edu).

### Materials availability

This study did not generate new unique reagents.

### Data and code availability

All code developed for this publication is available in the GitHub repository TargetOrtho2_Python3 at https://github.com/jdrumley1989/TargetOrtho2_Python3 (https://doi.org/10.5281/zenodo.14750829).

## Acknowledgments

We would like to thank Zhenying Tian for writing the first version of the R script TargetOrthoFIMO_motif_search_terminal.R and for further work in developing the R scripts. We would also like to thank Zhenying Tian and Daniel M. Merritt for their help in troubleshooting the installation of TargetOrtho2. We would like to thank Marion Boeglin and Surojit Sural for allowing us to test the installation and use of TargetOrtho2 on their computers. We would like to thank Lori Glenwinkel for providing instructions and code for updating the genome versions and adding new genomes to TargetOrtho2. We would like to thank Itai Toker for providing genome sequence and annotations for *Caenorhabditis tropicalis* to add this genome to TargetOrtho2. This work was funded by the Howard Hughes Medical Institute (O.H.) and by a BRAIN Initiative NRSA F32 fellowship from the National Institute of Neurological Disorders and Stroke (F32MH136667; J.D.R.). J.H.K. was funded by grant R35GM131746 from the National Institute of General Medical Sciences (P.I. Iva Greenwald).

## Author contributions

Conceptualization, O.H.; methodology, J.D.R., J.H.K., and O.H.; investigation, J.D.R. and J.H.K.; formal analysis, J.D.R. and J.H.K.; writing – original draft, J.D.R.; writing – review and editing, J.D.R., J.H.K., and O.H.; supervision, O.H.; funding acquisition, J.D.R. and O.H.

## Declaration of interests

The authors declare no competing interests.

## Declaration of generative AI and AI-assisted technologies in the writing process

During the preparation of this work, J.H.K. used ChatGPT4o in order to write the python script execute_copy.py. J.D.R. also used ChatGPT4o for troubleshooting the conversion of targetortho.py to Python 3 code, the installation of TargetOrtho2 on Windows and Ubuntu systems, and updating and adding new genomes to TargetOrtho2. After using this, the authors reviewed the content as needed and take full responsibility for the content of the published article.

## References

[1] Glenwinkel L., Taylor S.R., Langebeck-Jensen K., Pereira L., Reilly M.B., Basavaraju M., Rafi I., Yemini E., Pocock R., Sestan N. (2021). In silico analysis of the transcriptional regulatory logic of neuronal identity specification throughout the C. elegans nervous system. Elife.

[2] Glenwinkel L., Wu D., Minevich G., Hobert O. (2014). TargetOrtho: A Phylogenetic Footprinting Tool to Identify Transcription Factor Targets. Genetics.

[3] Budirahardja Y., Tan P.Y., Doan T., Weisdepp P., Zaidel-Bar R. (2016). The AP-2 Transcription Factor APTF-2 Is Required for Neuroblast and Epidermal Morphogenesis in Caenorhabditis elegans Embryogenesis. PLoS Genet..

[4] Cornwell A.B., Zhang Y., Thondamal M., Johnson D.W., Thakar J., Samuelson A.V. (2024). The C. elegans Myc-family of transcription factors coordinate a dynamic adaptive response to dietary restriction. GeroScience.

[5] Masoudi N., Tavazoie S., Glenwinkel L., Ryu L., Kim K., Hobert O. (2018). Unconventional function of an Achaete-Scute homolog as a terminal selector of nociceptive neuron identity. PLoS Biol..

[6] Weinberg P., Berkseth M., Zarkower D., Hobert O. (2018). Sexually Dimorphic unc-6/Netrin Expression Controls Sex-Specific Maintenance of Synaptic Connectivity. Curr. Biol..

[7] Berghoff E.G., Glenwinkel L., Bhattacharya A., Sun H., Varol E., Mohammadi N., Antone A., Feng Y., Nguyen K., Cook S.J. (2021). The Prop1-like homeobox gene unc-42 specifies the identity of synaptically connected neurons. Elife.

[8] Kratsios P., Pinan-Lucarré B., Kerk S.Y., Weinreb A., Bessereau J.-L., Hobert O. (2015). Transcriptional Coordination of Synaptogenesis and Neurotransmitter Signaling. Curr. Biol..

[9] Vidal B., Gulez B., Cao W.X., Leyva-Díaz E., Reilly M.B., Tekieli T., Hobert O. (2022). The enteric nervous system of the C. elegans pharynx is specified by the Sine oculis-like homeobox gene ceh-34. Elife.

[10] Reilly M.B., Tekieli T., Cros C., Aguilar G.R., Lao J., Toker I.A., Vidal B., Leyva-Díaz E., Bhattacharya A., Cook S.J. (2022). Widespread employment of conserved C. elegans homeobox genes in neuronal identity specification. PLoS Genet..

[11] Sural S., Hobert O. (2021). Nematode nuclear receptors as integrators of sensory information. Curr. Biol..

[12] Bailey T.L., Boden M., Buske F.A., Frith M., Grant C.E., Clementi L., Ren J., Li W.W., Noble W.S. (2009). MEME Suite: tools for motif discovery and searching. Nucleic Acids Res..

[13] Bailey T.L., Johnson J., Grant C.E., Noble W.S. (2015). The MEME Suite. Nucleic Acids Res..

[14] Neph S., Kuehn M.S., Reynolds A.P., Haugen E., Thurman R.E., Johnson A.K., Rynes E., Maurano M.T., Vierstra J., Thomas S. (2012). BEDOPS: high-performance genomic feature operations. Bioinformatics.

[15] Quinlan A.R., Hall I.M. (2010). BEDTools: a flexible suite of utilities for comparing genomic features. Bioinformatics.

[16] Grant C.E., Bailey T.L., Noble W.S. (2011). FIMO: scanning for occurrences of a given motif. Bioinformatics.

[17] Howe K.L., Bolt B.J., Cain S., Chan J., Chen W.J., Davis P., Done J., Down T., Gao S., Grove C. (2016). WormBase 2016: expanding to enable helminth genomic research. Nucleic Acids Res..

[18] Howe K.L., Bolt B.J., Shafie M., Kersey P., Berriman M. (2017). WormBase ParaSite − a comprehensive resource for helminth genomics. Mol. Biochem. Parasitol..

[19] Sternberg P.W., Van Auken K., Wang Q., Wright A., Yook K., Zarowiecki M., Arnaboldi V., Becerra A., Brown S., Cain S. (2024). WormBase 2024: status and transitioning to Alliance infrastructure. Genetics.

[20] Yoshida K., Rödelsperger C., Röseler W., Riebesell M., Sun S., Kikuchi T., Sommer R.J. (2023). Chromosome fusions repatterned recombination rate and facilitated reproductive isolation during Pristionchus nematode speciation. Nat. Ecol. Evol..

[21] International Helminth Genomes Consortium (2019). Comparative genomics of the major parasitic worms. Nat. Genet..

[22] Nitta K.R., Jolma A., Yin Y., Morgunova E., Kivioja T., Akhtar J., Hens K., Toivonen J., Deplancke B., Furlong E.E.M., Taipale J. (2015). Conservation of transcription factor binding specificities across 600 million years of bilateria evolution. Elife.

[23] Mercatelli D., Scalambra L., Triboli L., Ray F., Giorgi F.M. (2020). Gene regulatory network inference resources: A practical overview. Biochim. Biophys. Acta. Gene Regul. Mech..

